# Monocyte Distribution Width Differentiates Bacterial Enterocolitis from Acute Severe Ulcerative Colitis in the Emergency Department

**DOI:** 10.1007/s10620-026-09786-w

**Published:** 2026-03-02

**Authors:** Christopher F. D. Li Wai Suen, Shipraa Kaul, Ethan X. Z. Tan, Danny Con, Michelle Taylor, Joanne Wiid, Chris Hogan, Matthew C. Choy, Kumar Visvanathan, Peter De Cruz

**Affiliations:** 1 Department of Medicine (Austin Health), The University of Melbourne, Melbourne, VIC Australia; 2 Department of Gastroenterology, Austin Health, Melbourne, VIC Australia; 3 Immunology Research Centre, St Vincent’s Hospital Melbourne, Melbourne, VIC Australia; 4 Department of Laboratory Haematology, Austin Health, Melbourne, VIC Australia; 5 Department of Medicine (St Vincent’s Hospital), The University of Melbourne, Melbourne, VIC Australia; 6 Present Address: Department of Pathology, Eastern Health, Melbourne, VIC Australia

**Keywords:** Biomarker, Diagnosis, Ulcerative colitis, Enterocolitis, Gastroenteritis

## Abstract

**Purpose:**

Differentiating bacterial enterocolitis from acute severe ulcerative colitis (ASUC) is a common diagnostic problem. Monocytes play a role in the pathogenesis of ulcerative colitis and show variation in size, which is measurable as monocyte distribution width (MDW). We aimed to assess whether MDW can differentiate bacterial enterocolitis from ASUC and predict therapeutic response in ASUC.

**Methods:**

We conducted a retrospective cohort study comprising three patient groups: ASUC, bacterial enterocolitis, and controls at a tertiary Australian center. MDW, routine biomarkers and clinical outcomes were recorded. Primary outcomes included the difference in MDW between patient groups and the performance of MDW in distinguishing ASUC from bacterial enterocolitis. Secondary outcomes included the prediction of treatment response in ASUC and the performance in identifying biochemical remission post-ASUC.

**Results:**

176 patients were identified (53 ASUC, 70 bacterial enterocolitis and 53 controls). At presentation, patients with bacterial enterocolitis had the highest MDW (median 23.6, IQR 20.7–25.8) compared to ASUC (19.0, IQR 17.9–21.2; *P* < 0.001) and controls (16.8, IQR 15.9–18.0; *P* < 0.001). MDW discriminated bacterial enterocolitis from ASUC (Area under the curve [AUC]: 0.78, 95% CI: 0.70–0.87, *P* < 0.001). In ASUC, MDW correlated with CRP and fecal calprotectin. MDW on the day of infliximab administration predicted infliximab response (AUC: 0.80, 95% CI: 0.61–1.0, *P* = 0.002). Three months post-ASUC, MDW identified biochemical remission (fecal calprotectin < 100 µg/g; AUC 0.80, 95% CI: 0.64–0.95, *P* < 0.001).

**Conclusion:**

MDW is a novel biomarker that may help distinguish ASUC from bacterial enterocolitis. In patients with ASUC, MDW may predict inpatient infliximab response and identify biochemical remission at 3 months. Validation is required to confirm its utility.

**Supplementary Information:**

The online version contains supplementary material available at 10.1007/s10620-026-09786-w.

## Introduction

Ulcerative colitis (UC) is characterized by relapse and remission. Biomarkers may help distinguish active disease from remission and may help distinguish flares of UC from infective exacerbations. In addition, biomarkers may help predict response to therapy and may be particularly helpful in acute severe ulcerative colitis (ASUC) where both disease severity and response to therapy are highly variable [1, 2].

Monocyte distribution width (MDW) is a hematological parameter that can be generated by newer generation analysers on routine full blood examination (FBE). MDW reflects variation in monocyte size and is elevated during monocyte activation in response to infection. MDW has been proposed as a tool to aid early sepsis diagnosis in the emergency department (ED) [3–5] and may have prognostic value in patients with Covid-19 [6]. The role of MDW in inflammatory disorders such as inflammatory bowel disease has yet to be assessed. Distinguishing bacterial enterocolitis from flares of UC is challenging as both typically present with diarrhea, with both often associated with C-reactive protein (CRP) elevation [7, 8]. Diagnostic tests including fecal culture and multiplex polymerase chain reaction (PCR) used to differentiate the two entities often have a turnaround time of several days, potentially delaying treatment.

Given the greater magnitude of inflammatory response we frequently observe in bacterial enterocolitis compared to ASUC, we hypothesized that patients presenting with bacterial enterocolitis would have a higher MDW compared to both controls and patients with ASUC.

We further postulated that MDW would be elevated in patients with ASUC and decrease to levels similar to controls in the setting of remission. We also hypothesized that MDW would correlate with established markers of disease activity and help predict response to medical therapy.

The primary aim of our study was to assess whether MDW at the time of presentation can distinguish patients with ASUC from those presenting with bacterial enterocolitis. The secondary aims were to assess whether MDW can function as a marker of disease activity and remission in UC, and whether it is associated with treatment response in ASUC.

## Methods

### Study Design and Population

We conducted a retrospective cohort study at a tertiary hospital in Melbourne, Australia. We included three patient groups:

1. *ASUC group* patients presenting to ED with ASUC, treated with intravenous steroids and/or infliximab rescue therapy.
2. *Bacterial enterocolitis group *patients presenting to ED with enterocolitis from a bacterial pathogen.
3. *Control group* patients without active inflammation comprising outpatients with chronic hepatitis B in the immune control phase.

### Case Identification

#### ASUC Group

Adult patients admitted with ASUC between April 2020 and May 2022 were identified from hospital medical records using ICD-10-AM codes, based on Truelove and Witt’s criteria. We excluded patients with Crohn’s disease and patients with UC who had concomitant bacterial enterocolitis. As we were interested in assessing response to infliximab, we excluded patients who were already on an advanced therapy.

#### Bacterial Enterocolitis Group

Adults presenting to ED with diarrhea due to bacterial enteritis and/or colitis were identified using ICD-10-AM codes. Only cases confirmed by stool culture or multiplex PCR were included. Patients with other infections (e.g., pneumonia, urinary tract infections) were excluded to avoid confounding MDW elevation.

#### Control Group

Adults with chronic hepatitis B in immune control phase were identified from an outpatient clinic database (viral load < 2000 IU/mL, HBeAg negative, anti-HBe positive, and normal ALT; males: < 40 U/L, females: < 35 U/L). Those on antiviral therapy were excluded.

### Management of Patients with ASUC

Patients with ASUC received intravenous hydrocortisone 100 mg 6-hourly, with steroid response assessed between days 3 and 7 using the Travis score [9, 10]. Steroid responders were discharged on a tapering course of oral prednisolone starting at 40 mg daily. Steroid non-responders received 1–3 inpatient doses of infliximab (5 mg/kg or 10 mg/kg), at the treating clinician’s discretion, up to a maximum inpatient dose of 20 mg/kg.

Response to the infliximab rescue dose(s) was defined as ≤ 4 bowel actions/day, with a reduction in rectal bleeding and CRP < 15 mg/L. Infliximab responders were discharged on tapering oral prednisolone and completed infliximab induction as outpatients. Non-responders received tofacitinib sequential therapy, and colectomy was considered for patients who failed these treatments. Patients were followed up in the Inflammatory Bowel Disease outpatient clinic.

### Monocyte Distribution Width

MDW measurements have been routinely performed on FBE samples processed at our center since April 2020 using a DxH 900 Hematology Analyzer (Beckman Coulter, Inc; v2.0.14 software). The analyser reports cell differentials, monocyte cell volumes and their distribution (monocyte distribution width [MDW]). While MDW is FDA and CE-approved for sepsis detection[5], it remains a research parameter at our center; hence, results are not made available to clinicians and did not influence patient management.

### Data Collection

Data were obtained from hospital records and pathology databases, including demographics, MDW, and routine laboratory results. For the ASUC group, response to intravenous steroids, need for infliximab rescue, and response to infliximab were recorded. Biochemical remission at 3 months was defined as fecal calprotectin < 100 µg/g.

### Outcomes

Primary outcomes were the difference in MDW at presentation between ASUC, control, and bacterial enterocolitis groups, and the performance of MDW at distinguishing ASUC from bacterial enterocolitis. Secondary outcomes included correlations between MDW and established disease activity markers across the whole cohort and in ASUC. In ASUC, the performance of MDW at predicting treatment response and identifying biochemical remission at follow-up was also evaluated.

### Statistical Methods

Nonparametric continuous variables were expressed as medians with interquartile range (IQR). Categorical variables were expressed as frequencies with percentages. Comparisons of variables between groups were performed using analysis of variance or t-tests for continuous variables and Chi-square tests for categorical variables. Spearman’s rank correlation rho was used to determine associations between biomarkers. The discriminative performance of biomarkers was assessed using logistic regression as well as receiver operating characteristic (ROC) curve analysis and the area under the curve (AUC). The accuracy of biomarkers including the sensitivity, specificity, positive predictive value (PPV) and negative predictive value (NPV) were reported based on biomarker thresholds as determined by Youden’s index. Statistical analyses were performed using RStudio version 2023.06.1 + 524.

### Ethical Considerations

The study complied with the Declaration of Helsinki and was approved by the Institutional Research Ethics Committee (HREC/88449). Need for informed consent was waived due to the low-risk, retrospective design using de-identified data.

## Results

### Patient Demographics and Routine Biomarkers

Our cohort consisted of a total of 176 patients, including 53 in the ASUC group, 70 in the bacterial enterocolitis group, and 53 in the control group.

Baseline characteristics at ED presentation are summarized in Table 1*.* Two-way comparisons between the ASUC and bacterial enterocolitis groups are shown in Supplementary Table S1 while pathogens isolated in patients with bacterial enterocolitis are summarized in Supplementary Table S2.

**Table 1:** Baseline characteristics comparing controls with ASUC and bacterial enterocolitis at time of ED presentation (*n* = 176). Comparisons made using analysis of variance for continuous variables and chi-square test for categorical variables

Variable, median (IQR)	Controls (*n* = 53)	ASUC (*n* = 53)	Bacterial enterocolitis (*n* = 70)	*P*
Age, years	61.7 (54.0–68.3)	33.8 (26.7–51.9)	63.0 (36.3–76.4)	< 0.001
Male, *n* (%)	30 (57)	25 (47)	25 (36)	0.07
MDW	16.8 (15.9–18.0)	19.0 (17.9–21.2)	23.6 (20.7–25.8)	< 0.001
RDW, fL	13.4 (13.0–14.0)	13.4 (12.9–14.4)	13.7 (13.0–14.8)	0.13
Hb, g/L	144.0 (137.0–149.0)	130.0 (121.0–138.0)	133.0 (120.2–147.5)	< 0.001
Hct, %	43.0 (40.0–45.0)	39.0 (35.0–41.0)	40.0 (36.0–44.0)	< 0.001
WCC, × 10^9^/L	5.3 (4.5–6.1)	9.6 (7.3–12.2)	9.8 (6.8–12.4)	< 0.001
Neutrophils, × 10^9^/L	2.8 (2.3–3.5)	7.4 (5.1–9.0)	7.2 (4.3–9.8)	< 0.001
Lymphocytes, × 10^9^/L	1.8 (1.4–2.1)	1.3 (0.9–1.9)	1.0 (0.7–1.5)	< 0.001
Monocytes, × 10^9^/L	0.4 (0.3–0.5)	0.8 (0.5–1.1)	0.8 (0.6–1.1)	< 0.001
Platelets, × 10^9^/L	199.0 (180.5–245.5)	312.0 (267.0–372.0)	222.0 (176.5–269.8)	< 0.001
NLR	1.5 (1.2–2.1)	5.2 (3.4–8.8)	6.7 (4.0–13.1)	< 0.001
PLR	119.5 (89.1–149.8)	220.7 (170.0–339.0)	227.8 (146.6–331.8)	< 0.001
CRP, mg/L	–	23.9 (6.6–60.9)	78.3 (21.2–129.5)	0.002
Albumin, g/L	40.0 (39.0–42.0)	34.0 (31.0–38.0)	36.0 (32.0–40.0)	< 0.001
CAR, mg/g	–	0.7 (0.2–2.0)	2.2 (0.9–3.6)	0.003

ASUC, acute severe ulcerative colitis; IQR, interquartile range; MDW, monocyte distribution width; RDW, red cell distribution width; WCC, white cell count; NLR, neutrophil-to-lymphocyte ratio; PLR, platelet-to-lymphocyte ratio; CRP, C-reactive protein; Hb, hemoglobin; Hct, hematocrit; CAR, CRP-to-albumin ratio

**Fig. 1:**
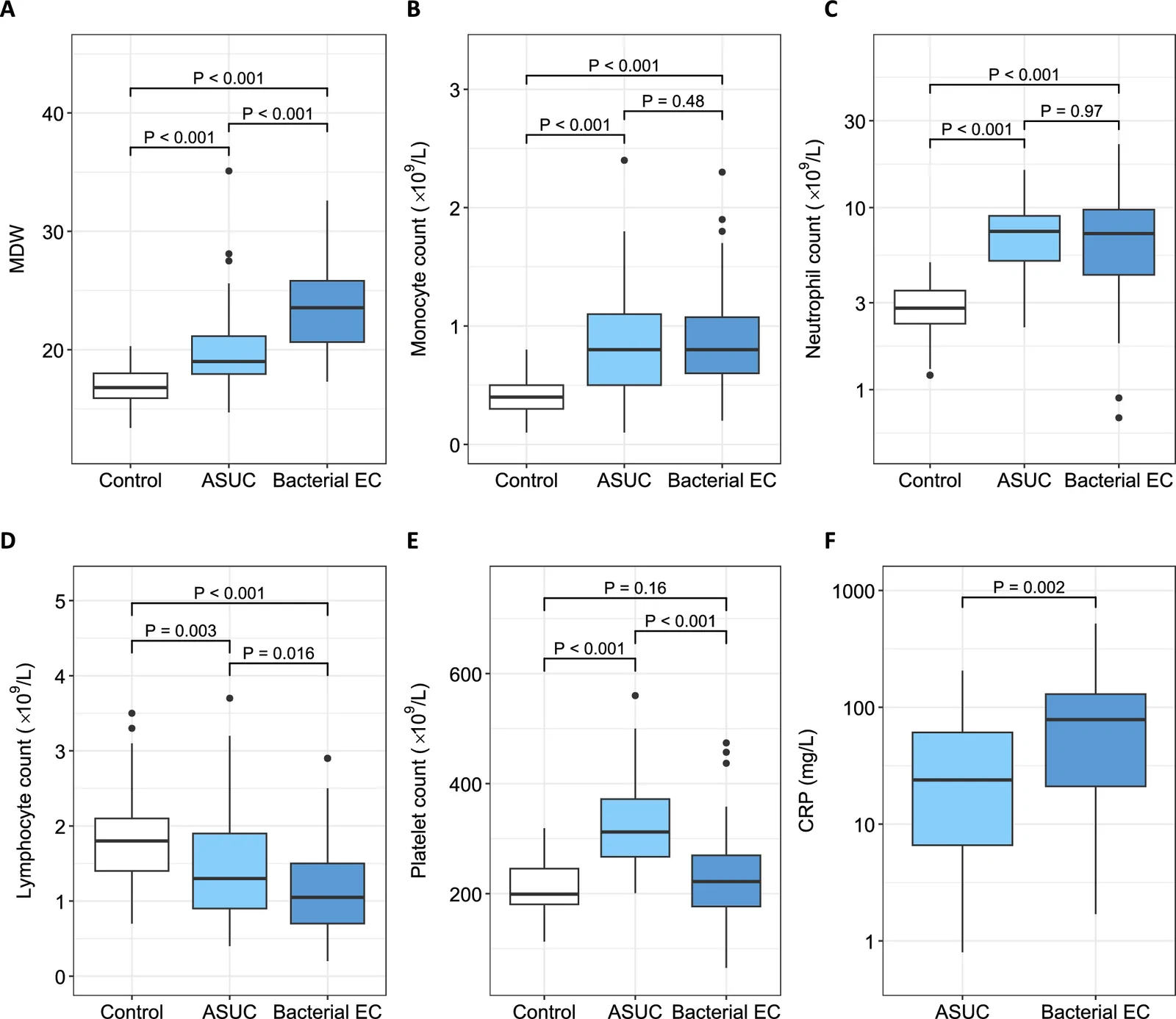
Box plots of baseline biomarkers comparing control, ASUC and bacterial enterocolitis (EC) groups (biomarkers taken at emergency presentation for ASUC and bacterial EC groups). **A** MDW; **B** monocyte count; **C** neutrophil count; **D** lymphocyte count; **E** platelet count; **F** C-reactive protein (CRP). Footnotes: ASUC, acute severe ulcerative colitis; Bacterial EC, bacterial enterocolitis; MDW, monocyte distribution width; CRP, C-reactive protein

Patients with ASUC were generally younger than controls and patients with bacterial enterocolitis. Although monocyte and neutrophil counts were elevated in both ASUC and bacterial enterocolitis compared to controls, they did not differentiate ASUC from bacterial enterocolitis. Lymphocyte count was lowest in bacterial enterocolitis compared to both ASUC and control, while platelet count was elevated in ASUC compared to control and bacterial enterocolitis. CRP was significantly higher in bacterial enterocolitis compared to ASUC *(*Fig. 1**A–F**).

### Assessment of MDW as a Biomarker

#### MDW at Presentation Across the Three Patient Groups

At time of presentation, patients with bacterial enterocolitis had the highest MDW (median 23.6, IQR 20.7–25.8) compared to patients with ASUC (19.0, IQR 17.9–21.2; *P* < 0.001) and controls (16.8, IQR 15.9–18.0; *P* < 0.001) *(*Fig. 1A*)*.

While patients with ASUC were generally younger compared to those with bacterial enterocolitis or controls, MDW did not correlate with age (spearman rho − 0.051, *P* = 0.50) and nor did sex (rho − 0.125, *P* = 0.10).

#### MDW Distinguishes Bacterial Enterocolitis from ASUC

On ROC analysis, MDW discriminated bacterial enterocolitis from ASUC with an AUC of 0.78 (95% CI 0.70–0.87; *P* < 0.001). After adjusting for age and sex, every one-point increase in MDW was associated with a 1.38-times increased odds of bacterial enterocolitis over ASUC (95% CI: 1.20–1.60). Using Youden’s index, a MDW threshold ≥ 22.3 had 64.3% sensitivity, 84.6% specificity, 63.8% NPV and 84.9% PPV for bacterial enterocolitis.

MDW (AUC 0.78) was superior to CRP (AUC: 0.70) in discriminating between bacterial enterocolitis and ASUC and had a similar performance to platelet count (AUC: 0.82) (Table 2).

**Table 2:** Performance of biomarkers taken at emergency presentation in discriminating bacterial enterocolitis from ASUC, with estimated unadjusted and adjusted odds ratios using logistic regression after controlling for age and sex (*n* = 123)

Predictor	AUC (95% CI)	Unadjusted			Adjusted	
		OR (95% CI)	*P*		OR (95% CI)	*P*
MDW	0.78 (0.70–0.87)	1.34 (1.18–1.53)	< 0.001		1.38 (1.20–1.60)	< 0.001
RDW, %	0.55 (0.45–0.66)	1.02 (0.88–1.18)	0.81		0.93 (0.78–1.10)	0.38
Hb, g/L	0.57 (0.47–0.67)	1.02 (1.00–1.04)	0.13		1.05 (1.02–1.08)	0.001
Hct, %	0.60 (0.50–0.70)	1.08 (1.00–1.17)	0.041		1.21 (1.09–1.35)	< 0.001
WCC, × 10^9^/L	0.48 (0.38–0.58)	0.82 (0.34–1.99)	0.66		0.92 (0.35–2.46)	0.88
Neutrophils, × 10^9^/L	0.50 (0.40–0.61)	0.86 (0.45–1.66)	0.66		0.88 (0.44–1.78)	0.72
Lymphocytes, × 10^9^/L	0.63 (0.53–0.73)	0.37 (0.17–0.79)	0.010		0.39 (0.17–0.92)	0.031
Monocytes, × 10^9^/L	0.54 (0.43–0.64)	1.59 (0.75–3.36)	0.23		1.92 (0.83–4.46)	0.13
Platelets, × 10^9^/L	0.82 (0.74–0.89)	0.99 (0.98–0.99)	< 0.001		0.99 (0.98–0.99)	< 0.001
NLR	0.58 (0.48–0.68)	1.39 (0.89–2.18)	0.15		1.35 (0.83–2.19)	0.23
PLR	0.45 (0.35–0.56)	0.74 (0.40–1.37)	0.34		0.62 (0.31–1.25)	0.18
CRP, mg/L	0.70 (0.58–0.81)	1.63 (1.17–2.27)	0.004		1.74 (1.19–2.53)	0.004
Albumin, g/L	0.58 (0.47–0.69)	1.06 (0.98–1.14)	0.134		1.12 (1.02–1.23)	0.013
CAR, mg/g	0.69 (0.57–0.81)	1.31 (1.03–1.68)	0.03		1.32 (1.01–1.72)	0.04

ASUC, acute severe ulcerative colitis; AUC, area under the curve; OR, odds ratio; CI, confidence interval; MDW, monocyte distribution width; RDW, red cell distribution width; WCC, white cell count; NLR, neutrophil-to-lymphocyte ratio; PLR, platelet-to-lymphocyte ratio; CRP, C-reactive protein; Hb, hemoglobin; Hct, hematocrit

#### Correlation Between MDW and Inflammatory Biomarkers

Combining the three patient groups (*n* = 176), MDW correlated positively with routine markers of inflammation including white cell count, neutrophil count, neutrophil-to-lymphocyte ratio, platelet-to-lymphocyte ratio, monocyte count, CRP and CRP-to-albumin ratio. Conversely, MDW correlated negatively with hemoglobin, hematocrit, lymphocyte count and albumin (Table 3; left panel).

**Table 3:** Spearman rank correlation of MDW with known inflammatory markers

Factor	All patients (*n* = 176)			ASUC patients (*n* = 53)	
	Rank correlation	*P*		Rank correlation	*P*
RDW	0.11	0.14		− 0.04	0.81
Hb	− 0.23	0.003		− 0.10	0.48
Hct	− 0.23	0.002		− 0.13	0.36
WCC	0.44	< 0.001		0.23	0.11
Neutrophils	0.47	< 0.001		0.19	0.17
Lymphocytes	− 0.38	< 0.001		− 0.13	0.36
Monocytes	0.39	< 0.001		0.12	0.40
Platelets	0.11	0.16		0.36	0.009
NLR	0.53	< 0.001		0.19	0.17
PLR	0.41	< 0.001		0.19	0.18
CRP^a^	0.72	< 0.001		0.58	< 0.001
Albumin	− 0.51	< 0.001		− 0.43	0.003
CAR	0.76	< 0.001		0.63	< 0.001
Fecal Calprotectin^b^	-	-		0.35	0.02

^a^CRP not performed in control group; *n* = 96 for rank correlation on left column. ^b^ Fecal calprotectin only performed in ASUC patients. MDW, monocyte distribution width; ASUC, acute severe ulcerative colitis; RDW, red cell distribution width; WCC, white cell count; NLR, neutrophil-to-lymphocyte ratio; PLR, platelet-to-lymphocyte ratio; CRP, C-reactive protein; Hb, hemoglobin; Hct, hematocrit; CAR, CRP-to-albumin ratio

*Left*: all patients. *Right*: Patients with ASUC

### Treatment Outcomes in the ASUC Cohort

Of the 53 patients with ASUC, 25 (47%) responded to intravenous steroids while 28 (53%) were steroid-refractory and required infliximab rescue. Of those, 24 responded to infliximab and 4 received sequential tofacitinib rescue. Two patients required colectomy at 3 months with a further patient needing colectomy 12 months after ASUC presentation.

### Assessment of MDW as a Biomarker in ASUC

#### Correlation Between MDW and Established Markers of Disease Activity in ASUC

To ascertain its potential usefulness as a biomarker in UC, we correlated MDW with established markers of activity.

In ASUC, MDW correlated positively with CRP (Spearman rank correlation, rho = 0.58, *P* < 0.001), platelet count (rho = 0.36, *P* = 0.009), fecal calprotectin (rho = 0.35, *P* = 0.02) and CRP-to-albumin ratio (rho = 0.63, *P* < 0.001), and correlated negatively with albumin (rho = − 0.43, *P* = 0.003) (Table 3-right panel).

#### Prediction of Response to Medical Therapy and Colectomy in ASUC

Given its correlation with routine disease activity markers, we evaluated the performance of MDW and other biomarkers in predicting steroid and infliximab response in ASUC and the need for colectomy.

MDW at presentation did not differ between steroid responders and non-responders (18.8, IQR: 17.5–20.9 vs 19.1, IQR: 18.2–21.9, *P* = 0.35) and did not predict steroid response (AUC: 0.58, 95% CI: 0.42–0.73, *P* = 0.35). In contrast, white cell count, neutrophil count, neutrophil-to-lymphocyte ratio, CRP and CRP-to-albumin ratio predicted response to intravenous steroids (Table 4-left panel).

**Table 4:** Performance of biomarkers in predicting outcomes in ASUC

Predictor	Predicting steroid response (*n* = 53)		Predicting infliximab response (baseline bloods) (*n* = 28)		Predicting infliximab response (day 0 IFX bloods) (*n* = 28)	
	AUC (95% CI)	*P*	AUC (95% CI)	*P*	AUC (95% CI)	*P*
MDW	0.58 (0.42–0.73)	0.35	0.76 (0.59–0.94)	0.003	0.80 (0.61–1.00)	0.002
RDW	0.55 (0.39–0.71)	0.57	0.60 (0.19–1.00)	0.63	0.66 (0.23–1.00)	0.47
Hb	0.53 (0.37–0.69)	0.71	0.62 (0.29–0.94)	0.49	0.58 (0.27–0.89)	0.62
Hct	0.54 (0.39–0.70)	0.59	0.68 (0.39–0.96)	0.22	0.59 (0.31–0.88)	0.52
WCC	0.70 (0.56–0.84)	0.007	0.68 (0.46–0.90)	0.10	0.79 (0.52–1.00)	0.034
Neutrophils	0.72 (0.58–0.86)	0.003	0.73 (0.50–0.96)	0.047	0.78 (0.48–1.00)	0.07
Lymphocytes	0.60 (0.45–0.76)	0.19	0.49 (0.23–0.75)	0.94	0.56 (0.22–0.90)	0.74
Monocytes	0.58 (0.42–0.74)	0.33	0.70 (0.46–0.94)	0.10	0.68 (0.40–0.96)	0.22
Platelets	0.56 (0.39–0.72)	0.49	0.55 (0.25–0.84)	0.76	0.75 (0.57–0.93)	0.008
NLR	0.71 (0.57–0.85)	0.004	0.54 (0.27–0.82)	0.77	0.59 (0.26–0.93)	0.58
PLR	0.62 (0.46–0.77)	0.14	0.52 (0.25–0.79)	0.88	0.57 (0.22–0.93)	0.69
CRP	0.76 (0.61–0.90)	0.001	0.86 (0.69–1.00)	< 0.001	0.94 (0.84–1.00)	< 0.001
Albumin	0.58 (0.42–0.75)	0.33	0.75 (0.25–1.00)	0.33	0.72 (0.43–1.00)	0.13
CAR	0.72 (0.55–0.89)	0.012	0.85 (0.54–1.00)	0.025	0.92 (0.82–1.00)	< 0.001
FCP (admission)	0.61 (0.43–0.79)	0.22	0.56 (0.23–0.89)	0.73	–	–

ASUC, acute severe ulcerative colitis; AUC, area under the curve; OR, odds ratio; CI, confidence interval; MDW, monocyte distribution width; RDW, red cell distribution width; WCC, white cell count; NLR, neutrophil-to-lymphocyte ratio; PLR, platelet-to-lymphocyte ratio; CRP, C-reactive protein; Hb, hemoglobin; Hct, hematocrit; FCP, fecal calprotectin; CAR, CRP-to-albumin ratio

*Left*: biomarkers taken at emergency presentation predicting steroid response. *Middle*: biomarkers taken at time of emergency presentation (baseline) predicting infliximab response. *Right*: biomarkers taken on day 0 prior to infliximab administration predicting infliximab response

Following intravenous steroids, MDW decreased to a median of 17.6 (IQR: 16.3–19.9) in responders compared to 19.4 (IQR: 17.6–22.0) in non-responders (*P* = 0.028) (Supplementary Figure S1).

In steroid-refractory patients, both a lower MDW at the time of presentation (AUC: 0.76, 95% CI: 0.59–0.94; *P* = 0.003) (Table 4*-*middle panel) and a lower MDW on the day of (but prior to) infliximab administration predicted infliximab response (AUC: 0.80, 95% CI: 0.61–1.00, *P* = 0.002) (Table 4*-*right panel). In addition, white cell count, platelet count, CRP, and CRP-to-albumin ratio measured on the day of infliximab administration predicted infliximab response (Table 4*-*right panel).

MDW at presentation predicted 3-month colectomy (AUC 0.72, *P* < 0.001); however, this should be interpreted with caution as only two patients underwent colectomy by month 3.

#### Change in MDW Following Treatment

To assess the responsiveness of MDW as a biomarker in ASUC, we compared MDW at admission and discharge. In patients with ASUC, MDW decreased from a median of 19.5 (IQR: 17.4–23.4) at presentation to 17.6 (IQR: 16.3–18.7; *P* < 0.001) after a median length of stay of 7 days (IQR 5–9) (Supplementary Figure S2).

#### MDW at Follow-up After Presentation with ASUC

Follow-up data was available for 40 patients after a median of 85 days (IQR: 71–148) following their presentation with ASUC. Twenty-six were in biochemical remission (fecal calprotectin < 100 µg/g) while 14 had active disease (fecal calprotectin ≥ 100 µg/g).

Follow-up MDW in patients with UC in biochemical remission did not differ from controls (18.0, IQR: 16.9–18.9 vs 16.8, IQR: 15.9–18.0, *P* = 0.08).

**Fig. 2:**
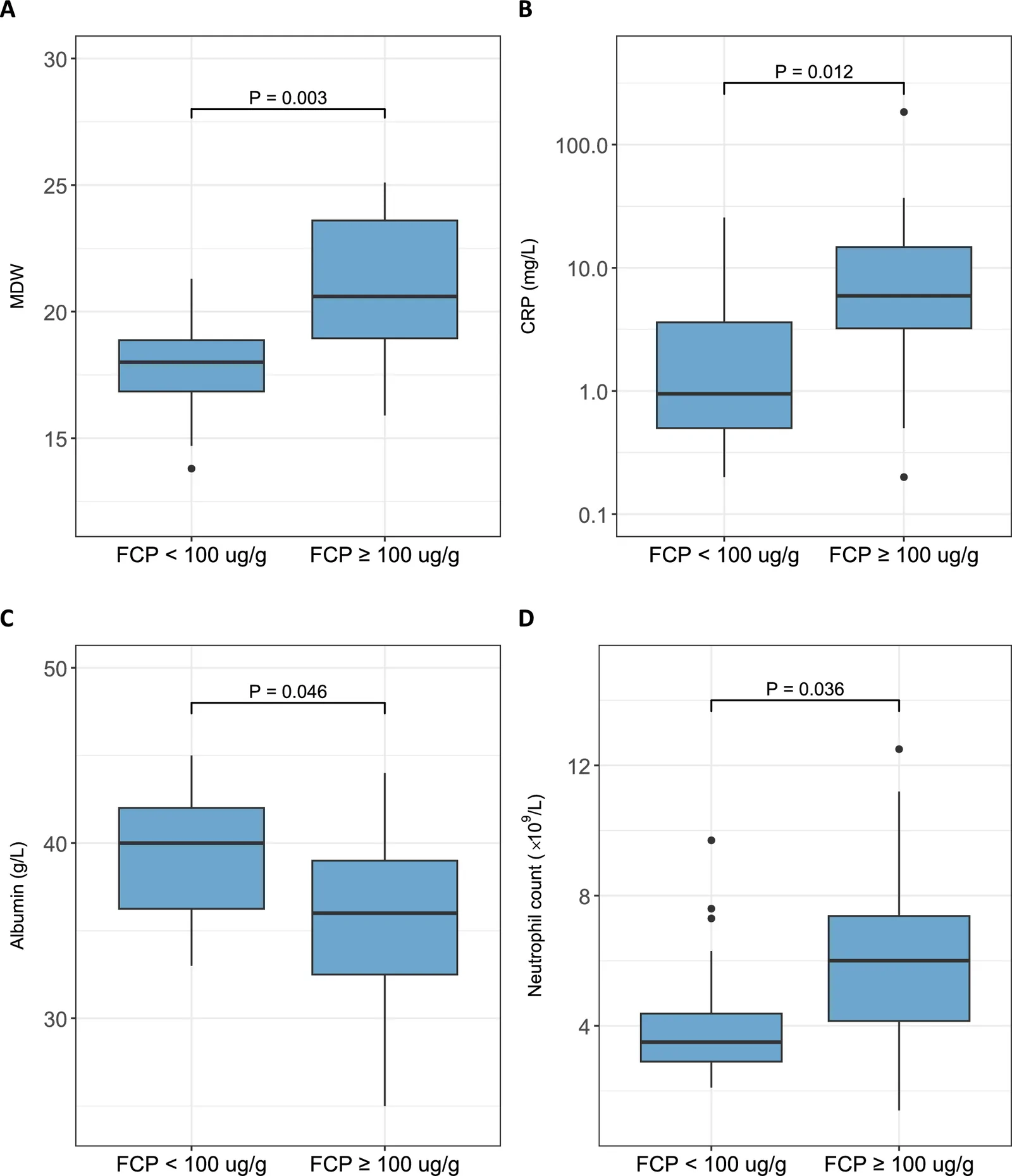
Biomarkers in patients with ulcerative colitis in biochemical remission (fecal calprotectin [FCP] < 100 ug/g) vs those with biochemically active disease (FCP ≥ 100 ug/g) after a median of 85 days following admission with acute severe ulcerative colitis (ASUC)

Patients with UC in biochemical remission had lower MDW (18.0, IQR 16.9–18.9 vs 20.6, IQR 19.0–23.6, *P* = 0.003), lower CRP (1.0 mg/L, IQR 0.5–3.6 vs 6.2 mg/L, IQR 3.3–14.9, *P* = 0.012), lower neutrophil count (3.5 × 10^9^/L, IQR 2.9–4.4 vs 6.0 × 10^9^/L, IQR 4.2–7.4, *P* = 0.036), and higher albumin (40 g/L, IQR 36–42 vs 36 g/L, IQR 33–39, *P* = 0.046) compared to those with biochemically active disease (Fig. 2A–D).

MDW predicted biochemical remission at the same time point (AUC 0.80, 95% CI 0.64–0.95, *P* < 0.001). A MDW threshold of <18.7 at follow-up had 71% sensitivity, 79% specificity, 85% PPV and 61% NPV in identifying those who were in biochemical remission. MDW outperformed white cell count, neutrophil count, neutrophil-to-lymphocyte ratio, CRP, albumin, and CRP-to-albumin ratio in predicting biochemical remission (Table 5).

**Table 5:** Follow-up data of ASUC patients (*n* = 40) after a median of 85 days following admission with ASUC, showing discriminative performance of biomarkers at follow-up for biochemical remission (fecal calprotectin < 100 µg/g) and Spearman rank correlations with fecal calprotectin as a continuous variable (with *P* value for rank correlations)

Predictor	AUC (95% CI)	Spearman rank correlation	*P* (correlation)
MDW	0.80 (0.64–0.95)	0.54	< 0.001
RDW	0.60 (0.41–0.79)	0.25	0.13
Hb	0.70 (0.53–0.87)	− 0.26	0.11
Hct	0.73 (0.56–0.89)	− 0.29	0.07
WCC	0.70 (0.51–0.89)	0.43	0.005
Neutrophils	0.71 (0.51–0.90)	0.48	0.002
Lymphocytes	0.50 (0.30–0.71)	− 0.03	0.84
Monocytes	0.59 (0.39–0.78)	0.31	0.06
Platelets	0.60 (0.41–0.79)	0.12	0.47
NLR	0.63 (0.43–0.82)	0.35	0.029
PLR	0.56 (0.36–0.77)	0.14	0.38
CRP	0.75 (0.57–0.92)	0.45	0.003
Albumin	0.69 (0.52–0.87)	− 0.44	0.004
CAR	0.75 (0.58–0.92)	0.46	0.003

ASUC, acute severe ulcerative colitis; AUC, area under the curve; OR, odds ratio; CI, confidence interval; MDW, monocyte distribution width; RDW, red cell distribution width; WCC, white cell count; NLR, neutrophil-to-lymphocyte ratio; PLR, platelet-to-lymphocyte ratio; CRP, C-reactive protein; Hb, hemoglobin; Hct, hematocrit, CAR: CRP-to-albumin ratio

## Discussion

Our study is the first to study MDW, a novel inflammatory biomarker, in UC and suggests that MDW may be useful for both inpatient and outpatient management. MDW helps differentiate bacterial enterocolitis from ASUC, correlates with conventional markers of biochemical disease activity, and predicts response to rescue therapy in ASUC.

MDW measured at ED presentation was able to differentiate bacterial enterocolitis from ASUC. Distinguishing bacterial enterocolitis from ASUC in patients presenting with diarrhea is a common diagnostic dilemma as diagnosis via stool culture or PCR testing can take several days. Yet, early diagnosis is important to help more accurately target an antibacterial versus anti-inflammatory therapeutic approach. Reasons for the higher MDW observed in bacterial enterocolitis compared to ASUC may relate to the higher systemic inflammatory response (e.g., fever and tachycardia) often observed in bacterial enterocolitis along with the potentially greater magnitude of monocyte activation by bacterial pathogens in the setting of infection. Hence, MDW may be a helpful early diagnostic biomarker in differentiating bacterial enterocolitis from ASUC.

MDW was elevated in active UC and correlated with established biochemical markers of disease activity, CRP and fecal calprotectin. The mechanistic explanation for MDW’s elevation likely relates to monocyte and/or macrophage activation in UC pathogenesis [11–14]. Disruption in colonic tight junctions may also play a role, resulting in increased bowel wall permeability, which exposes the gut immune system (including macrophages and monocytes) to more luminal antigens. While fecal calprotectin normalization is a useful biomarker that correlates with mucosal healing, barriers to its more widespread use include the inconvenience of patients having to collect their own stool and costs. Our study highlights the potential role of MDW, a blood-based biomarker, in identifying patients in biochemical remission (fecal calprotectin < 100 µg/g) in the outpatient setting. While MDW may not fully replace fecal calprotectin, it may augment clinical practice by giving patients access to a more acceptable and convenient test. Furthermore, as MDW can be measured on routine FBE by new generation blood analyzers, it entails no extra cost to the patient. Hence, MDW could be considered a cost-effective adjunctive biochemical biomarker of disease activity in UC.

Although MDW at presentation was not associated with steroid failure in our ASUC cohort, MDW taken on the day of infliximab rescue predicted infliximab response, suggesting that it may have potential as a predictive biomarker in ASUC. Validation studies are required to confirm whether MDW can be used to identify patients at high risk of infliximab non-response. The ability to identify such high-risk patients could enable clinicians to either intensify infliximab dosing, consider sequential therapy with cyclosporin or a Janus kinase inhibitor, or refer for colectomy.

Our study has several strengths. The large sample size comprising three distinct patient groups enabled comparison across ASUC, bacterial enterocolitis, and controls, which established an MDW threshold that can help differentiate bacterial enterocolitis from ASUC.

Our study also comprehensively assessed the utility of MDW as a novel biomarker in UC. By carefully characterizing the clinical outcomes of patients presenting with ASUC, we examined the utility of MDW as: (1) a biomarker of disease severity; (2) a predictive marker of infliximab response; and (3) a marker of biochemical remission in the outpatient setting.

Another strength of our study is the fact that MDW was first measured on routine FBE at the time of ED presentation, before institution of medical therapy (corticosteroid or antibiotic therapy) that could have altered MDW results, thus making MDW an attractive pre-treatment diagnostic biomarker.

Finally, blinding of clinicians from MDW results was maintained throughout the study. While MDW measurements are performed routinely on FBE at our center, the results are not released by our clinical pathology department. As such, MDW did not influence clinical management, thus removing the risk of performance or detection bias.

Our study is limited by its retrospective nature. Although ASUC patients were younger than those with bacterial enterocolitis and controls, MDW was not influenced by age. Furthermore, MDW discriminated bacterial enterocolitis from ASUC both before and after adjusting for age and sex.

Due to the challenges involved in obtaining blood samples of healthy controls in a retrospective study, our control group comprised patients with chronic hepatitis B in the immune control phase attending routine outpatient follow-up. Nonetheless, we believe that these patients were similar to healthy controls given their suppressed viral loads and normal liver biochemistry indicating the absence of active hepatic inflammation.

This study was conducted at a single tertiary center. While the findings are encouraging, validation in an independent cohort is required before routine clinical adoption of MDW to exclude bacterial enterocolitis and facilitate management of UC. Although in this cohort MDW was slightly superior to CRP at differentiating bacterial enterocolitis from ASUC and at predicting month 3 biochemical remission post-ASUC, future studies should determine whether MDW provides additional clinical benefit over existing inflammatory biomarkers such as CRP. Nonetheless, given that the pathogenesis of UC involves monocyte trafficking to the gut, it is biologically plausible that MDW may confer additional benefit over existing biomarkers.

Finally, while MDW effectively discriminated between bacterial enterocolitis and ASUC, patients with UC and concomitant bacterial enterocolitis were excluded from our study due to few such patients and to avoid confounding. We postulate that in such cases, MDW levels would be elevated to levels seen in bacterial enterocolitis as the latter constitutes a condition with higher inflammatory activity. Further studies could examine MDW specifically in patients with UC presenting with superimposed bacterial enterocolitis.

A major clinical appeal of MDW as a biomarker is the fact that it can be obtained on routine FBE on newer analysers without the need for additional processing, cost, or local expertise. Its rapid turnaround time makes it especially attractive in the emergency setting and potentially in outpatient settings where fecal calprotectin results may not be available. To our knowledge, however, MDW measurement is based on a proprietary technology currently only available on DxH 900 and DxH 690 T Beckman Coulter analysers [15]. Therefore, while future routine clinical use of MDW in UC management is feasible, its widespread adoption will depend on broader availability of MDW-capable analysers in the clinical laboratory setting.

In conclusion, this study is the first to evaluate the potential role of MDW, an inflammatory biomarker, in ASUC. MDW can help discriminate bacterial enterocolitis from ASUC and may help improve patient care by prompting early initiation of empirical antibiotics. In addition, we identified MDW as a novel biomarker of disease activity in UC, with potential utility in identifying patients at risk of failing infliximab rescue therapy in ASUC. While our findings are encouraging, validation studies are required to confirm our results and to determine whether MDW provides any additional benefit beyond existing biomarkers.

## Supplementary Information

Below is the link to the electronic supplementary material.

Supplementary file1 (PDF 214 KB)

## Data Availability

Data sharing is not available as participants of this study did not provide written consent for their data to be shared publicly. Ethics was approved as part of a low-risk ethics submission.
