# Supplementary material for: Monocyte Distribution Width Differentiates Bacterial Enterocolitis from Acute Severe Ulcerative Colitis in the Emergency Department
Source: Dig Dis Sci. 2026 Mar 2;71(8):3291–301. doi: 10.1007/s10620-026-09786-w (PMC13500438; doi:10.1007/s10620-026-09786-w)
Supplement: Supplementary file 1 [file 10620_2026_9786_MOESM1_ESM.pdf]

Supplementary information

Supplementary Tables

Supplementary Table S1. Comparison of patient characteristics and biomarkers at time of emergency presentation in the ASUC and bacterial gastroenteritis groups.

Variable, median (IQR)	ASUC (*n*=53)	Bacterial enterocolitis (*n*=70)	*P*
Age, years	33.8 (26.7-51.9)	63.0 (36.3-76.4)	<0.001
Male, *n* (%)	25 (47)	25 (36)	0.20
MDW, IU	19.0 (17.9-21.2)	23.6 (20.7-25.8)	<0.001
RDW, fL	13.4 (12.9-14.4)	13.7 (13.0-14.8)	0.34
WCC, $\times 10^9$/L	9.6 (7.3-12.2)	9.8 (6.8-12.4)	0.73
Neutrophils, $\times 10^9$/L	7.4 (5.1-9.0)	7.2 (4.3-9.8)	0.97
Platelets, $\times 10^9$/L	312.0 (267.0-372.0)	222.0 (176.5-269.8)	<0.001
Lymphocytes, $\times 10^9$/L	1.3 (0.9-1.9)	1.0 (0.7-1.5)	0.016
NLR	5.2 (3.4-8.8)	6.7 (4.0-13.1)	0.12
PLR	220.7 (170.0-339.0)	227.8 (146.6-331.8)	0.38
CRP, mg/L	23.9 (6.6-60.9)	78.3 (21.2-129.5)	0.002
Hb, g/L	130.0 (121.0-138.0)	133.0 (120.2-147.5)	0.19
Hct, %	39.0 (35.0-41.0)	40.0 (36.0-44.0)	0.059
Monocytes, $\times 10^9$/L	0.8 (0.5-1.1)	0.8 (0.6-1.1)	0.48
Albumin, g/L	34.0 (31.0-38.0)	36.0 (32.0-40.0)	0.16
CAR, mg/g	0.7 (0.2-2.0)	2.2 (0.9-3.6)	0.003

Footnotes: ASUC, acute severe ulcerative colitis; IQR, interquartile range; MDW, monocyte distribution width; RDW, red cell distribution width; WCC, white cell count; NLR, neutrophil-to-lymphocyte ratio; PLR, platelet-to-lymphocyte ratio; CRP, C-reactive protein; Hb, haemoglobin; Hct, haematocrit; CAR, CRP-to-albumin ratio

Supplementary Table S2. Pathogens isolated from the 70 patients with bacterial enterocolitis

**Pathogen**	***n***
Campylobacter[a]	40
C. difficile	21
Salmonella	5
Shiga toxin-producing *E.Coli* [a]	3
Shigella/Enteroinvasive *E.Coli*	2

Footnotes: *C. difficile, Clostridioides difficile. E.Coli, Escherichia coli*. [a]One patient tested positive for both *Shiga toxin-producing E.coli and Campylobacter*

Supplementary Figures

Supplementary Figure S1. Box plots demonstrating dynamics of MDW in ASUC patients before and after intravenous corticosteroids, stratified by steroid response.

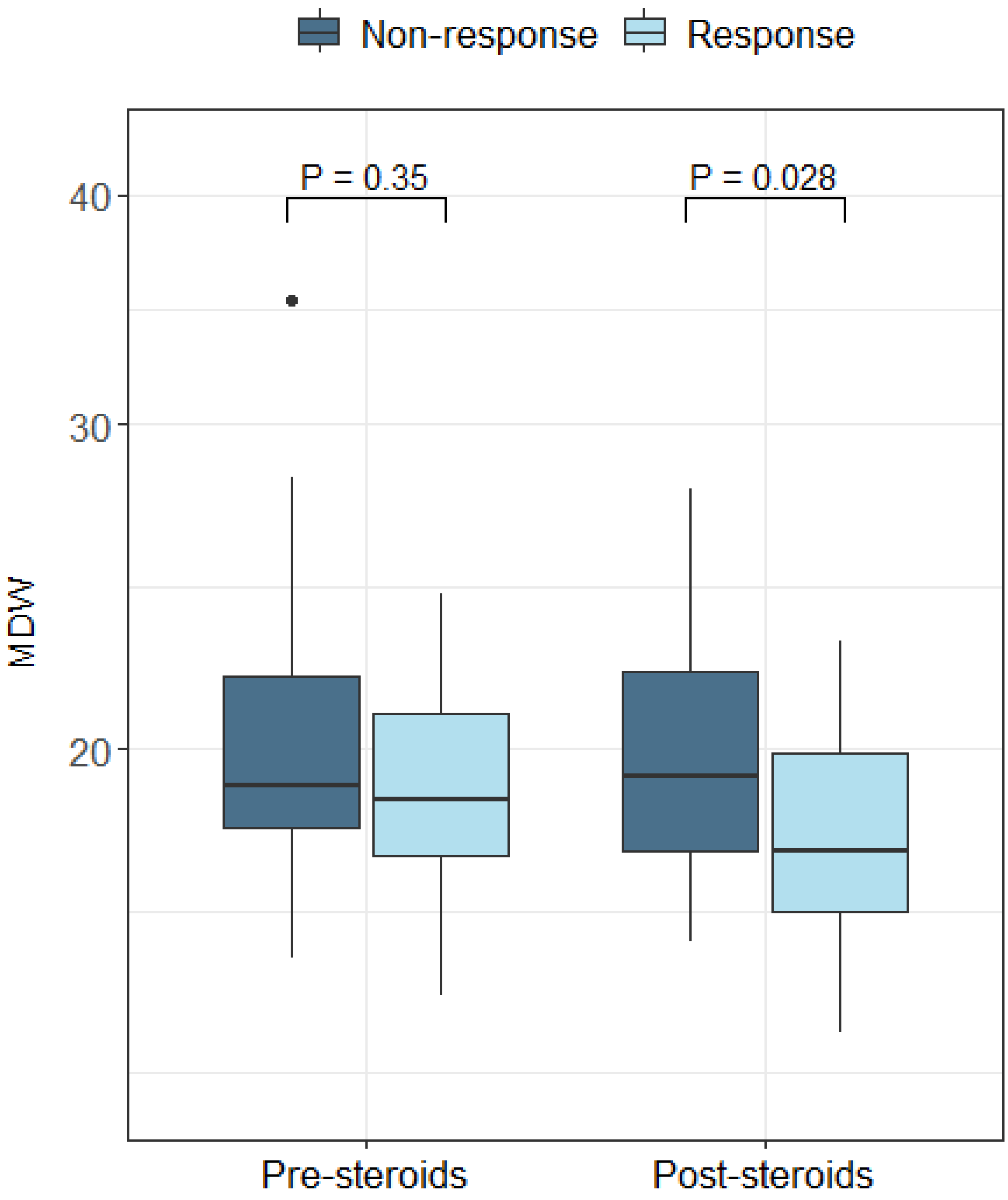

Footnotes: ASUC, acute severe ulcerative colitis; MDW, monocyte distribution width; steroids: IV corticosteroids (hydrocortisone).

Supplementary Figure S2. Box plots of MDW in ASUC patients at presentation and discharge.

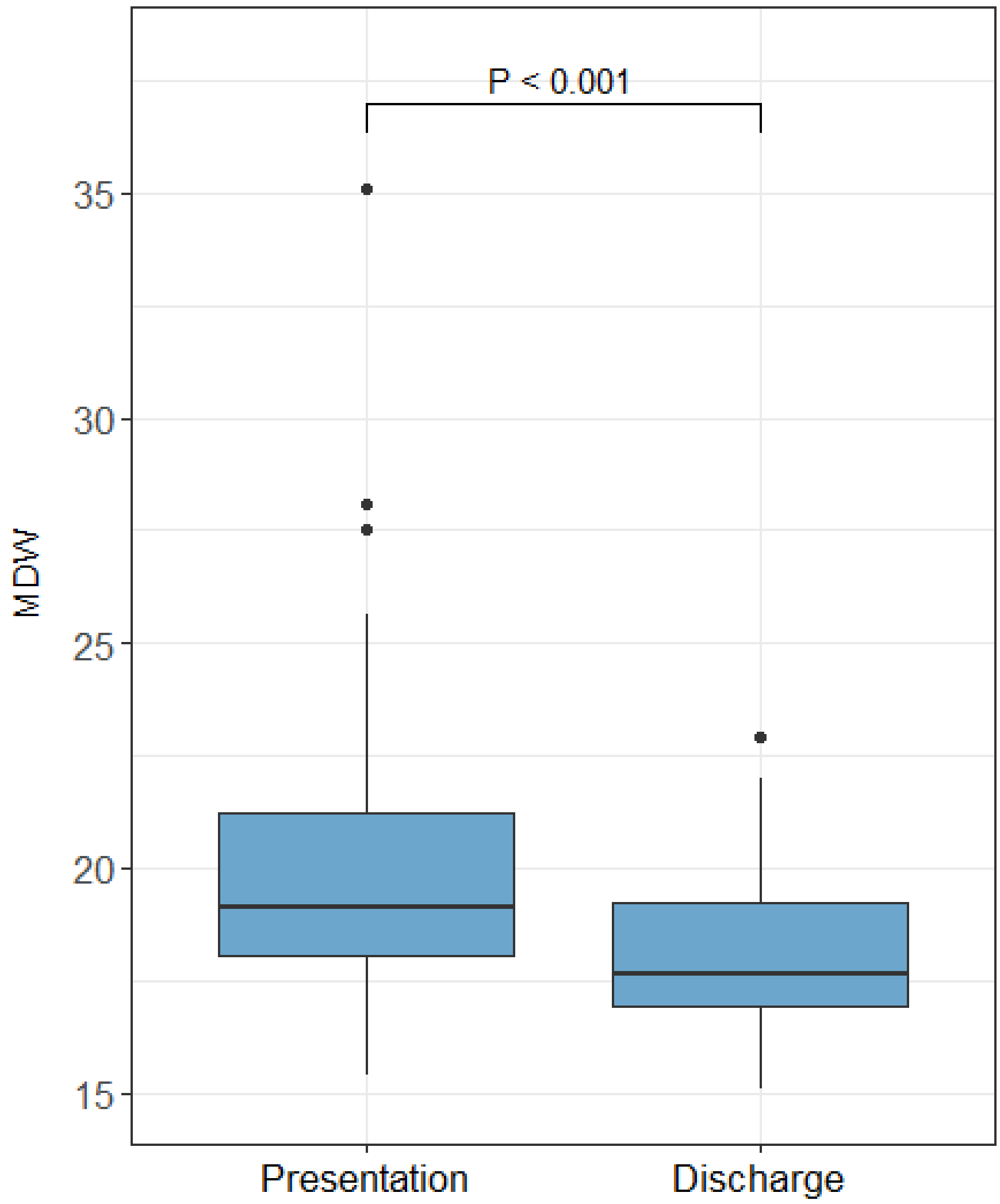

Footnotes: ASUC, acute severe ulcerative colitis; MDW, monocyte distribution width.
